# Training in lung cancer surgery through the metaverse, including extended reality, in the smart operating room of Seoul National University Bundang Hospital, Korea

**DOI:** 10.3352/jeehp.2021.18.33

**Published:** 2021-12-31

**Authors:** Huilyung Koo

**Affiliations:** JTBC Studios, Seoul, Korea; Hallym University, Korea

## Non-face to face training during the COVID-19 pandemic era

The coronavirus disease 2019 (COVID-19) pandemic has made it difficult to provide medical training across borders. In particular, it has become virtually impossible to see high-tech medical equipment from other countries and observe surgery to learn. However, educational methods involving the “metaverse” are also being introduced in the medical field to meet the growing demand for non-face-to-face education, as medical staff from around the world who visited Korea to gain expertise in medical technology and medical students who need to practice have had fewer opportunities to work with patients directly due to COVID-19. Efficient surgical training is also challenging using available video-conferencing systems such as Zoom. In order to resolve these limitations, new digital methods using metaverse technology are emerging in the Korean medical community (Supplement 1). In this editorial, I would like to introduce a training course in the metaverse held in Korea and describe expectations about how this technology will be used in the medical field in the future.

## Example of training in lung cancer surgery through the metaverse

On May 29, 2021, the 6th Outreach program using an extended reality (XR) technology platform was held under the supervision of the Asian Thoracic Surgery Education Group at the 29th Online Conference of the Asian Heart and Thoracic Surgery Society (Supplement 1). More than 200 thoracic surgeons from Asian countries attended the Outreach program and received training. Surgeons from Manchester University Hospital in the United Kingdom and National University Hospital in Singapore also accessed the virtual environment to visit the system and had active discussions.

**Fig. 1.**
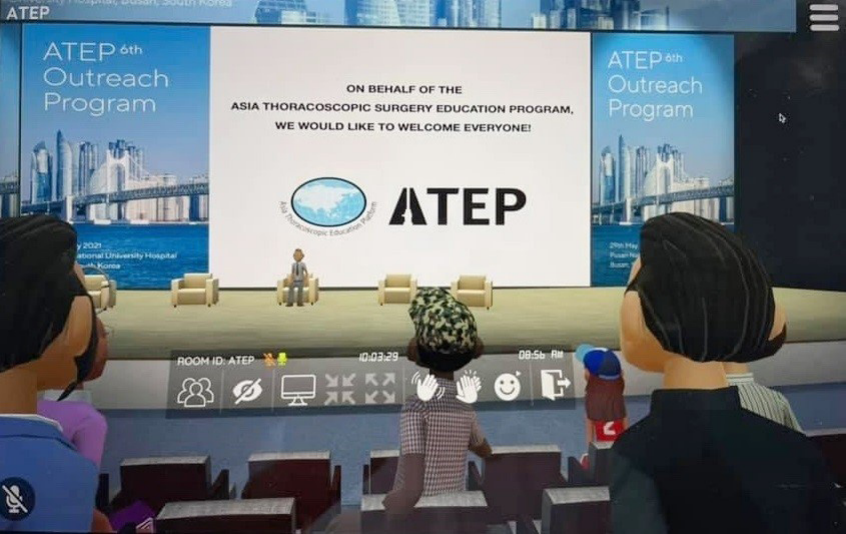
Avatars of participants in the virtual conference of the Asian Heart and Thoracic Surgery Society in 2021 (use of the photo was permitted by Seoul National University Bundang Hospital).

The participants in the program wore a head-mounted display (HMD) in their respective laboratories or experienced a real place in a virtual environment with their laptops. The 360° environment was implemented with HMDs and laptops through recent platform upgrades. After participants set up their individual avatars as in a game, they entered a virtual classroom where they viewed lectures on lung cancer surgery techniques and trends in virtual and mixed technology (Fig. 1). They also continued discussions in real-time while observing the surgical process in a virtual environment. Lung cancer surgery education was conducted with high-resolution virtual reality cameras broadcasting all surgical scenes in 360°. The virtual space was decorated to resemble a conference venue.

**Fig. 2.**
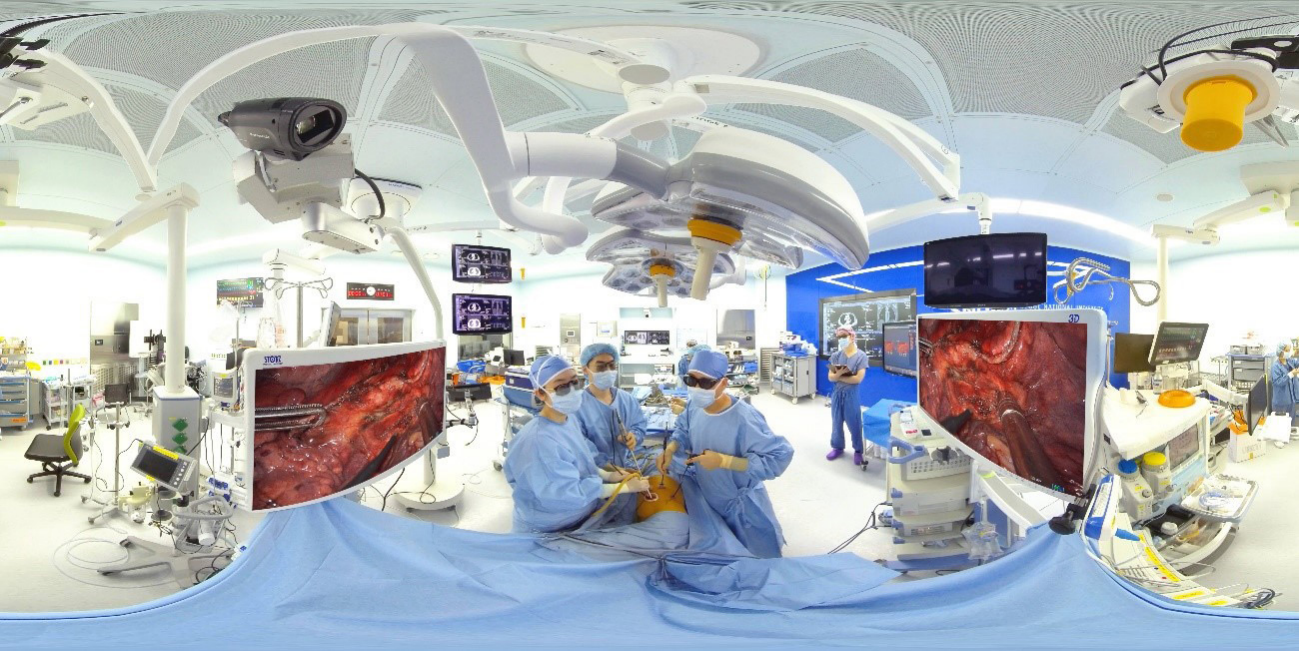
Surgical scene in the smart operating room, Seoul National University Bundang Hospital (use of the photo was permitted by Seoul National University Bundang Hospital).

**Fig. 3.**
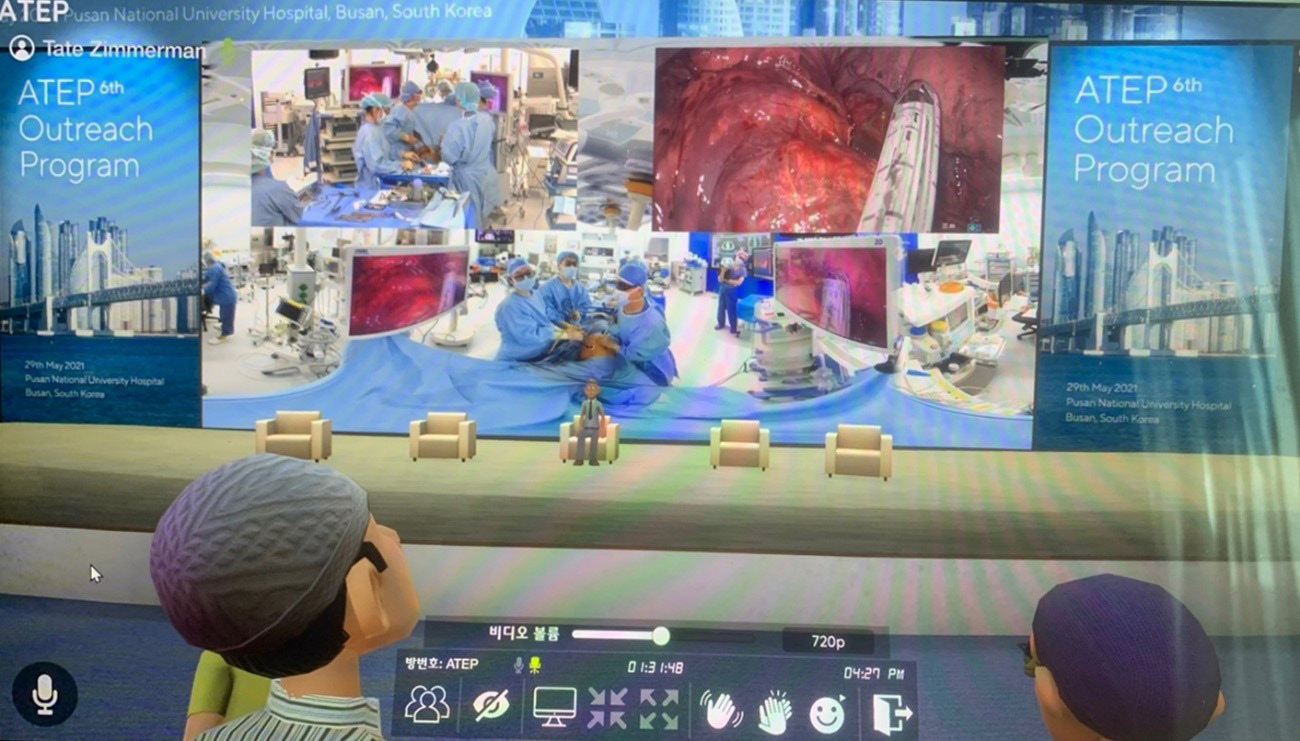
Avatars watching a surgical scene in the smart operating room, Seoul National University Bundang Hospital (use of the photo was permitted by Seoul National University Bundang Hospital).

Surgery was broadcast in the smart operating room of Seoul National University (SNU) Bundang Hospital. Participants indicated that it seemed like they were observing the procedure in the actual operating room because they could see the surgeon, the surgical nurse, and the environment in the operating room as desired through the 360°-8K-3D camera built in the operating room (Figs. 2, 3). The platform is characterized by a virtual environment and high-quality voice conversation through 3-dimensional (3D) XR immersive sound technology. Another advantage is that smooth real-time voice support and various screens such as the actual environment can be implemented. The surgical scene is more visible than when the observer enters the physical operating room and watches the procedure. Participants can also see the surgeon’s monitor and how the operating room nurses hand over surgical tools, how the forceps move, and even the sweat while they suture the wound. As participants move the mouse, they can see every corner of the operating room from the angle that the observer wants. If they wear a 3D headset, the visible scene changes every time they turn their heads, and the feeling of being in the operating room becomes more palpable than when a headset is not used.

## How could this training be accomplished?

Accomplishing this training was possible because SNU Bundang Hospital’s smart operating room and metaverse environment are combined. The term “metaverse” combines “meta,” which means “virtual and transcendent,” and “universe,” denoting the world [1]. The metaverse is a reproduction of reality in a virtual space. This concept can be intuitively understood by thinking of virtual spaces using avatars such as Roblox [2] and Fortnite [3]. SNU Bundang Hospital created a smart operating room in 2019 that contains 6 lenses in a virtual reality camera that shoots 360° in all directions, a high-resolution camera, and fluorescent imaging equipment that can visualize lymph nodes in 1 place in the operating room. Advanced imaging equipment has become critical with the increasing frequency of surgery to minimize laparotomy and leverage the power of endoscopy. Surgeons wear 3D glasses and observe high-resolution 3D images from laparoscopic cameras.

Using this technology, an observer can watch the process more extensively and precisely than when a surgeon opens the stomach and performs surgery. The operating room camera or lighting has a voice recognition function. A surgeon can operate with both hands and turn off the camera or light directly with a voice command. Medical staff may have a remote conversation about the biopsy results during surgery. For instance, if a surgeon removes tissue during surgery and sends it to a pathologist, it will be possible for the pathologist to display microscopic images on the large screen of the operating room after the biopsy. While watching the video, the surgeon would listen to the pathologic findings in real-time and share opinions. A control room where an observer can look into the smart operating room transparently is attached right next to it, allowing them to transmit videos or adjust cameras there.

## Importance of a smart operating room

After the COVID-19 pandemic, the importance of smart operating rooms has increased. Surgeons from the United States and Europe previously came to SNU Bundang Hospital to observe state-of-the-art surgery, but these visits have become impossible since COVID-19. However, through 360° video, surgeons located anywhere in the world can see the procedures as vividly as if they were in the operating room. There is no need to come to Korea. Dr. Sang-Hoon Ahn, a professor of surgery at SNU Bundang Hospital, said that “Video education using the metaverse is more effective than any other educational method available so far.” An observer can only see the abdominal cavity through a typical video broadcast, but the 360° video shows the entire situation in the operating room. For example, in Professor Ahn’s training on the use of articulated forceps for laparoscopic surgery, it is difficult to know precisely how to use the forceps only by looking at images of them moving in the abdominal cavity. However, through the 360° video, if Professor Ahn changes the wrist’s angle of movement, the observers can see how the forceps move inside the body. They can learn more accurately than from direct observation in the operating room [4]. Furthermore, the number of people who can physically enter an operating room is limited. In addition, in the case of endoscopic surgery, the monitor angle is tailored to the doctor who is operating, so even if someone enters the operating room, they often cannot see the surgical scene correctly. However, videos from the smart operating room do not have this drawback, and they have therefore been used for surgeons, nurses, and medical student education. Dr. So Hyun Kang, an SNU Bundang Hospital fellow, said that this educational system benefits medical residents: “Residents and fellows are always busy. I cannot run to the operating room to observe while working far away. It is only seen in medical dramas that medical doctors look down at the surgical scene from above.” Even if residents cannot watch a surgical broadcast in real-time, they can study by watching videos recorded from various angles later.

## Further application of the metaverse through tailored education and training

This demonstration at SNU Bundang Hospital is an example of using the metaverse in clinical education [4]. Among the various types of metaverse applications, this is an example of XR implemented by mixing augmented reality (AR) and virtual reality (VR). XR refers to all VR technologies ranging from VR to mixed reality and AR [5]. With the prolonged COVID-19 situation, the importance of medical education using smart operating rooms and the metaverse is growing. A technical leap is expected, such as higher camera resolution and the ability to deliver haptic input reflecting the surgeon’s tactile sensations. Thus, the metaverse is expected to be actively used in medical education or education for residents and students in the future. The use of the metaverse through tailored education and training in the COVID-19 era will be accelerated even in the post-COVID-19 period. Once these cutting-edge technologies are introduced into the medical field, they will contribute to the health of Koreans and people around the world.

## Supplementary materials

Data files are available from Harvard Dataverse: https://doi.org/10.7910/DVN/PNXOEK

Supplement 1. [Public report] Medical education in a virtual space, metaverse that is realistic.
