# Supplementary material for: Training in lung cancer surgery through the metaverse, including extended reality, in the smart operating room of Seoul National University Bundang Hospital, Korea
Source: J Educ Eval Health Prof. 2021 Dec 31;18:33. doi: 10.3352/jeehp.2021.18.33 (PMC8810683; doi:10.3352/jeehp.2021.18.33)

보도자료

SNUH 분당서울대학교병원

보도일시 : 배포 즉시 사용하시기 바랍니다.

**배 포 일**	2021년 5월 31일 (총 3매)		
**담당교수**	흉부외과<br>전상훈 교수	**담 당 자**	이제혁<br>김종섭, 김봄누리,<br>박여진, 정민제
**연 락 처**	-	**연 락 처**	031-787-1122
**첨부자료**	라이브서저리 외 관련 사진 3매		

분당서울대병원 흉부외과

의료 교육도 가상 공간에서… 실제 같은 메타버스

- 메타버스에 펼쳐진 수술실, 의료 교육 제한점 넘는다 -

- **5월 29일(토), 국내외 의료진 대상으로 XR기술 활용한 ATEP '제 6차 아웃리치 프로그램' 진행**
- **아바타로 소통하고, 가상의 강의실 접속해 실시간으로 강의 수강**
- **실제 수술장으로 들어가 바로 눈앞에서 수술 부위 확인하며 높은 몰입감과 현장감 느낄 수 있어**
- **코로나19로 인한 비대면 일상의 정착으로, 기존 원격 툴 한계 극복한 새로운 기술 의료현장에 도입**
- **교육뿐만 아니라, 진료·건강관리·디지털치료제의 검증까지 가능한 '가상의 종합병원' 구축이 목표**

의료 현장에서도 '메타버스' 교육 방식이 도입되고 있다. 세계 각국에서 의료 기술을 배우기 위해 한국을 찾던 해외 의료진은 물론 실습이 중요한 의대생들까지, 코로나19로 인해 직접 환자를 마주할 수 있는 기회가 줄어들면서 비대면 교육에 대한 수요가 커지고 있어서다.

줌과 같은 일반적인 화상 시스템으로는 효율적인 외과 수술교육이 어려운 게 사실이다. 이러한 제한점을 해결하기 위해 의료계에서도 XR 기술을 활용한 새로운 디지털 변화가 나타나고 있다. 메타버스 구현의 핵심기술이 바로 가상현실(VR)과 증강현실(AR), 혼합현실(MR)을 아우르는 확장현실(XR)이다.

지난 5월 29일, '2021년 아시아심장혈관흉부외과학회(ASCVTS) 제 29차 온라인 학술대회'에서 '아시아흉강경수술교육단(ATEP)' 주관으로 XR 기술 플랫폼을 활용한 '제 6차 아웃리치 프로그램(ATEP 6th Outreach Program)'이 진행됐다.

이번 아웃리치 프로그램에는 아시아 각국의 흉부외과 의료진 200여 명이 참석해 교육을 받았고, 영국 맨체스터대학병원과 싱가포르 국립대병원에서도 이 시스템을 견학하기 위해 가상환경에 접속해 활발한 토의를 나눴다. 작년 가을 베트남 흉부외과 의료진을 대상으로 처음 적용한데 이어, 이번에는 한 차원 높은 수준의 가상환경이 구현됐다.

프로그램 참석자들은 각자의 연구실에서 HMD(Head Mounted Display)를 착용하거나 노트북으로 현실 속 장소를 가상의 환경에서 그대로 체험했다. 최근 플랫폼 업그레이드를 통해 HMD 뿐만 아니라 노트북으로도 360도 환경이 구현된 덕분이다. 마치 게임처럼 본인의 아바타를 설정한 후 가상의 강의실에 입장해 폐암수술 기법과 가상융합기술 트렌드를 주제로 한 강의를 수강했고, 가상의 환경 속에서 수술 과정을 참관하며 실시간으로 토의를 이어갔다.

수술은 분당서울대병원 스마트수술실에서 중계됐다. 수술실에 구축된 360도-8K-3D카메라를 통해 집도의와 수술 간호사의 모습, 수술실 내 환경을 원하는 대로 볼 수 있어 실제 수술실 안에서 참관하는 것 같았다는 게 참석자들의 평가다.

해당 플랫폼은 가상환경 뿐만 아니라 3D XR 이머시브 사운드 기술을 통해 고품질의 음성 대화도 더욱 현장감 있게 할 수 있는 것이 특징이다. 원활한 실시간 음성지원과 실제 환경과 같은 다양한 화면 구현이 가능한 것 또한 장점이다.

향후 세계 유수의 병원들과 스마트 병원 연합체를 구성하여, 교육뿐만 아니라 진료 및 건강관리, 디지털 치료제의 검증 등을 실현할 수 있는 국경을 초월하는 '가상의 종합병원'을 구축하는 것이 궁극적인 목표다.

분당서울대병원 흉부외과 전상훈 교수(ASCVTS 회장·ATEP 설립자)는 "지난해부터 코로나19로 촉발된 이동 제한으로 메타버스 시대가 급물살을 탔다"며, "모든 산업분야가 그러하지만

특히 의료분야에서는 감염 우려 탓에 당장 대면 진료가 필요한 환자들이 어려움을 겪기도 하고, 실습이 중요한 의학교육에 있어 많은 어려움이 있다"고 말했다.

이어 전 교수는 "의료분야는 매우 복잡하고 진입장벽이 높지만, 그 가치는 다른 산업분야에 비해 상당히 크다"며, "단순한 VR 콘텐츠 몇 개를 제공하는 것을 넘어서서, 빅데이터·인공지능·5G 등 첨단 기술을 확장현실 기술과 융합한 '가상의 종합병원'을 구축해, 시공간을 초월하는 보다 상위 개념의 서비스를 통해 헬스케어 메타버스 시장을 선도적으로 이끌어가기 위해 노력할 것"이라고 덧붙였다.

한편, ATEP는 지난 2012년 국내를 비롯해 일본·중국·대만·싱가포르·태국·인도 등 아시아 지역 전역에 흉강경 수술 기법을 전파하고 체계적인 교육을 지원하고자 설립됐다. <끝>

[사진 설명]

[사진1]

분당서울대병원 스마트수술실에서 라이브 서저리를 시연하고 있는 분당서울대병원 흉부외과 전재현 교수 모습

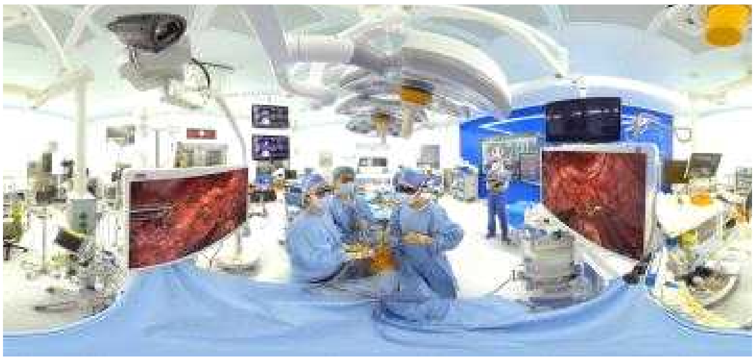

[사진2]

아바타 형태로 가상의 ATEP 아웃리치 프로그램 강의실에 입장한 모습(좌)

라이브 서저리 세션을 지켜보며 토의중인 아바타 형태의 참석자들 모습(우)

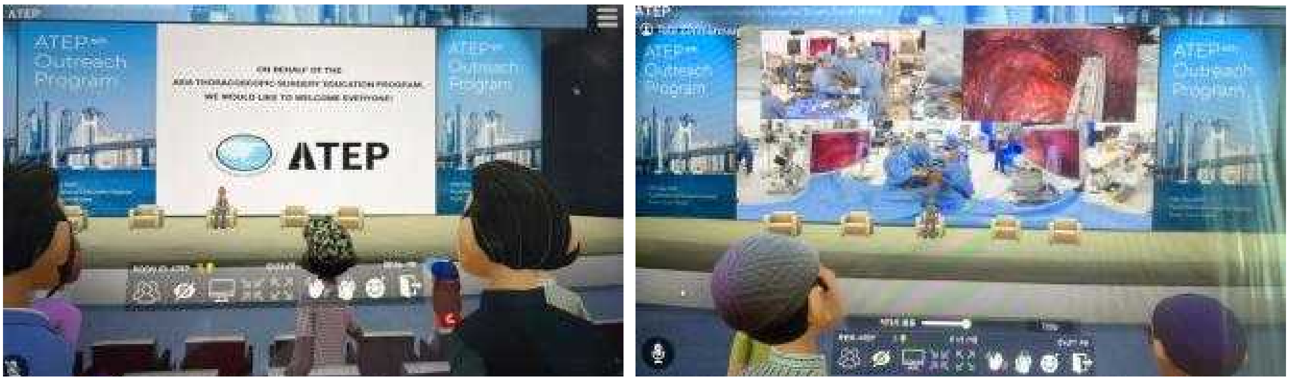

Supplement: Supplementary file 1 — Supplement 1. [Public report] Medical education in a virtual space, metaverse that is realistic. [file jeehp-18-33-suppl.pdf]
